# Periaortic Abscess following DeBakey Type-1 Aortic Dissection Repair with Dacron Graft—Early Diagnosis and Management

**DOI:** 10.1155/2019/6915356

**Published:** 2019-05-08

**Authors:** Rishi Raj, Dileep Unnikrishnan, Aasems Jacob, Kumar Ashish, Amulya Prakash, Ajay Shah

**Affiliations:** ^1^University of Kentucky, Lexington, KY, USA; ^2^Monmouth Medical Center, Long Branch, NJ, USA; ^3^Crozer-Chester Medical Center, Upland, PA, USA

## Abstract

A 71-year-old male with history of DeBakey type-1 aortic dissection and repair with dacron graft three months prior to presentation was brought to the emergency room with complaints of high-grade fevers, chills, and shortness of breath. Chest X-ray revealed right lower lobe infiltrates and widened superior mediastinum. A follow-up CT chest with contrast showed fluid collection around the aortic graft. He was started on intravenous broad-spectrum antibiotics, and a TEE was done for further evaluation of periaortic fluid collection which showed findings to suggest periaortic abscess. The patient underwent surgical drainage of the abscess and was found to have an abscess around the surgical aortic graft which was drained followed by two weeks of antibiotic treatment. The patient was discharged to a rehabilitation facility and remained asymptomatic at three-month follow-up appointment. Type-1 aortic dissection is a medical emergency requiring acute surgical intervention, and despite significant advancements in diagnosis and management, the immediate and long-term complications remain high leading to increased risk of mortality. Our patient developed spontaneous periaortic abscess three months postoperatively requiring intensive antibiotic therapy along with surgical drainage. Our case emphasizes the importance of early diagnosis and management of late complications of periaortic abscess in patients with aortic dissection repair.

## 1. Background

Graft and perigraft infections are dreaded complications following aortic repair surgeries with high mortality rates. Accurate and early detection and prompt surgical treatment are of paramount importance in reducing mortality in such cases. Although it has been widely accepted that surgical explantation with long-term antibiotics reduces mortality drastically compared to conservative measures, there is no consensus on the treatment approach for this entity. Recent studies have shown graft-sparing surgical therapy to be safe and effective for aortic graft infection which occur within 1 month postaortic dissection repair surgery. However, replacement of graft with biological conduits was showed to be better when aortic infection happens 3-6 months postaortic dissection repair [1]. Here, we present a case of early-onset periaortic infection and abscess formation following aortic dissection repair who was successfully managed without graft explantation.

## 2. Case Presentation

A 71-year-old Caucasian hypertensive, diabetic male with past medical history significant for critical coronary artery disease, hyperlipidemia, carotid artery stenosis, and subclavian vein thrombosis presented to the emergency room with chief complaints of high-grade fever of one day duration associated with chills and shortness of breath. Three months prior to presentation, the patient developed acute aortic dissection DeBakey type-1 for which he underwent emergent cardiothoracic surgery and dacron graft repair of the ascending aorta. Few days following his discharge, he started having low-grade fevers which was associated with night sweats, dry cough, and exertional dyspnea for which the patient did not seek medical attention until one week from the visit date. Prior to presentation, he was treated at an urgent-care facility with a five-day course of doxycycline for the same complaints with a working diagnosis of community-acquired pneumonia with only partial improvement of his symptoms. One day prior to presentation, he developed high-grade fever. He denied any chest pain, rashes on his body, and painful nodules on his hands or feet. On admission, his blood pressure was 137/68 mmHg, pulse rate 113 beats per minute, and respiratory rate 18/minute, and he was saturating of 99% on room air. He was febrile with temperature of 102.6°F. Physical examination was remarkable for tachycardia and early diastolic murmur in the aortic area and a pansystolic murmur best heard in the apex with radiation to the axilla. The sternotomy scar on his chest was well-healed.

**Figure 1:**
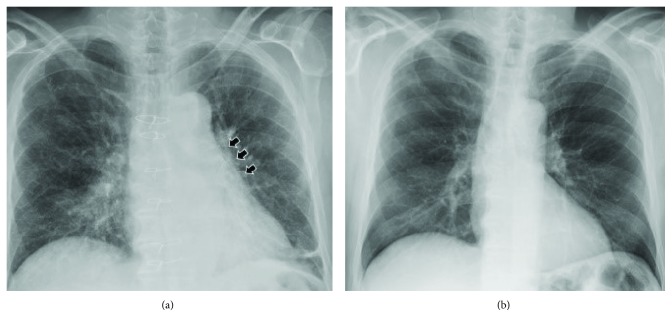
Chest X-ray on admission (a) compared to the prior chest X-ray (b); black arrows show superior mediastinal fullness compared to the prior study.

**Figure 2:**
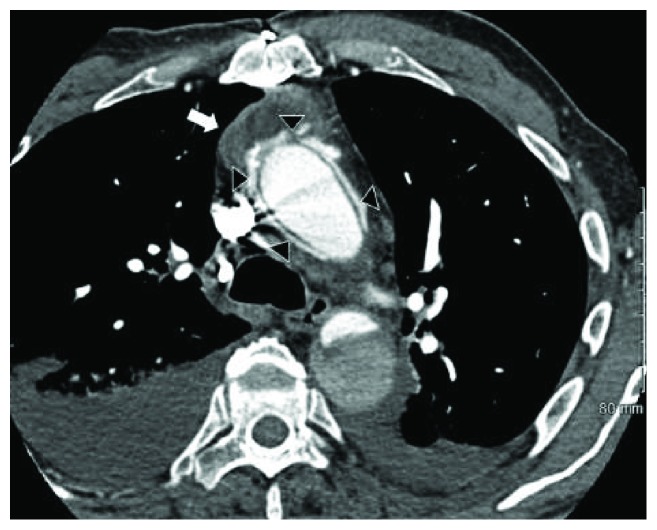
CT chest with contrast. White arrows show periaortic abscess in the ascending aorta.

**Figure 3:**
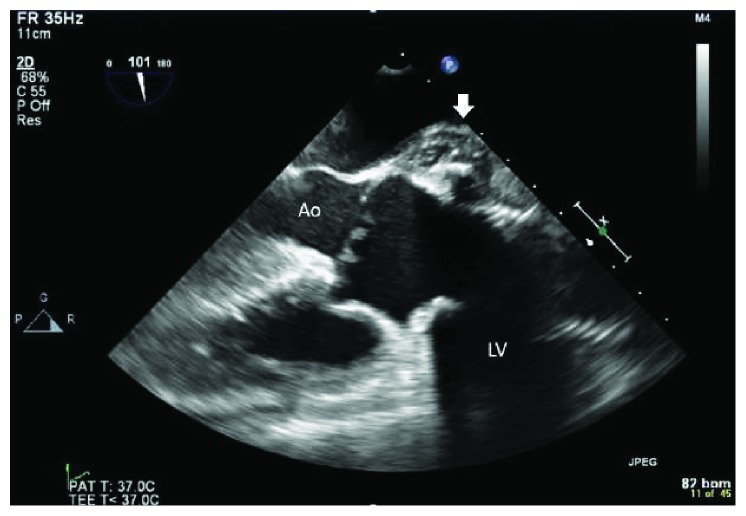
Mid-esophageal long-axis view of the heart with Doppler in the transesophageal echocardiogram showing severe aortic regurgitation.

**Figure 4:**
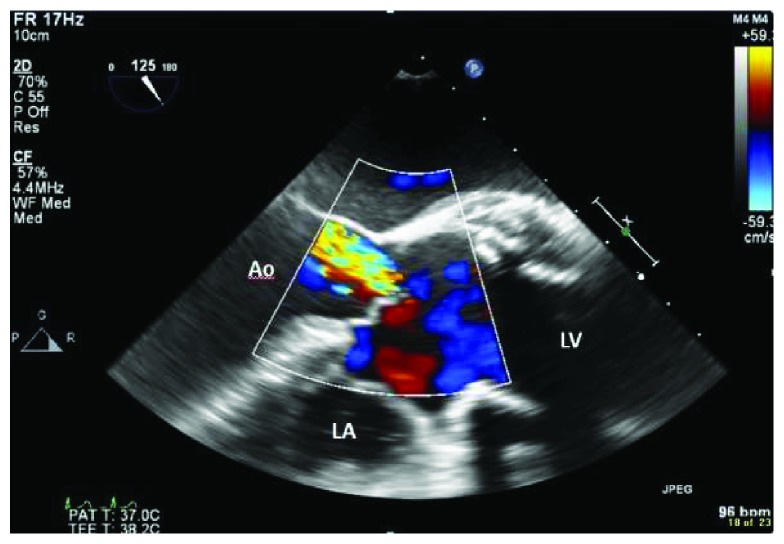
Mid-esophageal long-axis view of the heart in the transesophageal echocardiogram. White arrow shows periaortic abscess extending to the root of the aorta.

Initial laboratory investigation results are presented in Table 1, along with reference range values. Notably, his WBCs, lactic acid level, and procalcitonin levels were within normal range. A chest X-ray as part of the routine work-up was ordered from the emergency room and showed widening of the superior mediastinum compared to his prior film (Figure 1). Due to this concerning finding, a CT scan of the chest with contrast was ordered and showed a new fluid collection surrounding the ascending aorta and extending into the aortic arch measuring 5 cm in mediolateral dimension at the level of aortic arch (Figure 2). The patient was admitted to the medical floors with working diagnosis of aortic graft infection. Blood cultures were drawn and vancomycin 1 gm every 12 hours and piperacillin-tazobactam 4.5 g every 6 hours was started. Cardiothoracic surgeons were called in to evaluate the patient for possible surgical evacuation of the abscess and explantation of the graft. A transthoracic echocardiogram (TEE) was recommended by the surgical team for better visualization of the valves and their involvement. The TEE showed an echolucent area (Figure 3) consistent with fluid or blood around the ascending aorta conduit graft concerning for periconduit leak or abscess. It also revealed moderate-to-severe aortic regurgitation (Figure 4) with global left ventricular hypokinesis, but no valvular or perivalvular vegetations concerning for endocarditis. At this point, a decision was made to take the patient to the operating room for drainage of the abscess. On surgical exploration, purulent material was drained from the wall of the aorta around the graft and microbiological cultures were obtained. Intraoperatively, it was found that there was a clear demarcation between the graft and the abscess collection; and therefore, a decision was made not to explant the graft. Mediastinal drains were placed, and patient underwent continuous irrigation with betadine. Microbiological cultures from the abscess remained negative. Patient was continued on intravenous antibiotics for 2 weeks and was eventually discharge to rehabilitation after completion of two weeks of inpatient antibiotic treatment following sternotomy. He started to defervesce postoperatively and remained afebrile upon discharge. Interestingly, both his blood and pus cultures were negative, most likely because of early initiation of potent broad-spectrum antibiotics. The patient was advised to follow up with cardiology for aortic valve repair at a later date as an outpatient.

## 3. Discussion

Infections of the aortic graft are rare postoperative complications of aortic surgeries such as repair of the aortic dissection and aneurysms. Although the incidence varies from based on center and expertise ranging anywhere from 1-3% to 0.2-5% in various studies, the condition can be catastrophic and is associated with very high mortality rate ranging from 25 to 75% [2, 3]. Due to its rarity, there are no large studies identifying the predisposing factors for aortic graft infection. Immediate postoperative infections are considered to be from direct inoculation of microbial flora, whereas delayed infection might be due to unevacuated thrombus, persistent leak around graft, nosocomial septicemia, and/or immunocompromised states. Although source of graft infection following open or endovascular procedure remains unrecognized, skin flora is considered to be most commonly encountered pathogen. There are reports of duodenal defects leading to graft infections in cases of abdominal aortic aneurysm; however, no such direct seeding of microbial flora has been reported following thoracic aorta dissection repair.

In cases where microbiological culture remains negative due to use of antibiotics, molecular testing (including qPCR or broad-range 16S rDNA) could be useful as they do not rely on the patient being antibiotic naïve and can help establish infectious etiology. In addition, it would also help in differentiating more aggressive organism (e.g., *Staphylococcus aureus*) from less virulent pathogen and potentially indicated the source of infection as low-grade infection tends to happen at time of surgery. Although broad -range 16S rDNA PCR is very specific and have high positive and negative predictive values, it can detect all bacterial DNA present in a sample, including the contaminants which are unavoidably present in reagents, thereby increasing false-positive rate and decreasing sensitivity. It is therefore important to use qPCR studies as the first line and only consider broad-range 16S rDNA PCR as an adjunct to microbiological diagnostics as a second line when infection of a sterile site is highly suspected, but culture and qPCR for the most likely pathogens have been proven negative [4].

Symptoms of aortic graft infection are often vague, and this requires a high degree of suspicion on the clinician's side while treating patients with aortic grafts. It could present as a constellation of symptoms that include fever, malaise, weight loss, back pain, leukocytosis, or abdominal pain. Severe fulminant sepsis can be seen in about one-third of cases of early graft infections. Computerized tomography (CT) with contrast enhancement is the diagnostic modality of choice in aortic graft infections [5]. CT angiography can be supplemented by indium-leucocyte scanning and sinography, which could illustrate whether a draining sinus extends to the graft [6]. Findings to look for in CT scan includes the following:

- Persistence of perigraft fluid after 3 months of procedure.
- Presence of ectopic gas in the aortic wall.
- Increased soft tissue and loss of normal tissue planes.
- Formation of pseudoaneurysm.

Transthoracic or transesophageal echocardiography can support a CT with contrast findings in cases where the thoracic aorta is involved. A 2016 study by Lyons et al. elucidated the diagnostic criteria to be used in suspected cases of aortic graft infection [7]. The management of aortic graft infection collaboration (MAGIC) criteria includes clinical, radiological, and laboratory findings and categorizes each into three major and two minor criteria. Although there are no established treatment guidelines for this clinical entity due to its rarity, most of the existing literature endorses surgical removal of graft over conservative management alone. However, explantation of infected graft is not a good idea in very debilitated and patients with significant comorbidities. In case of conservative management, CT imaging-guided aspiration of the periaortic fluid can supplement antibiotic therapy. The choice of antibiotics is empirical until cultures are available and should have broad gram-positive coverage (both gram-positive and -negative in cases where aortoenteric fistula is suspected). There is no established consensus on the duration of treatment. Most published case reports opted for a minimum of 4-6 weeks of antibiotics with some cases resorting to lifelong antibiotic therapy [8]. Contraindication for conservative treatments are suture-line hemorrhage, graft-enteric fistula, and infected anastomotic aneurysm. High rate of mortality is the norm for aortic graft infection. As reported in an international enquiry with 62 reported cases from different international centers of vascular surgery, mortality among operative cases was 16.3% and that among conservative management was 36.4% [9]. With such high mortality rates, it is important to focus on prevention, early detection, and prompt treatment of this entity.

## 4. Conclusion

Aortic graft infection is a rare complication of postaortic repair surgeries with very high mortality. The presenting symptoms are often vague and depend on the time of presentation, part of the aorta that is infected, and the virulence of pathogen involved. Fever with new-onset murmur is red flag in patients with aortic grafts in situ and warrants further investigations. In addition, subtle findings such as widening of the mediastinum should not be neglected in such patients to ensure prompt diagnosis. CT chest with contrast enhancement is the diagnostic modality of choice which can reveal perigraft fluid, ectopic gas in the aortic wall, and loss of normal tissue. The criteria defined by MAGIC (Management of Aortic Graft Infection Collaboration) provides a handy tool to arrive at a diagnosis. Treatment with broad-spectrum antibiotics must be initiated at the earliest time after drawing blood cultures. Graft explantation with four to six weeks of antibiotics is the accepted practice in anyone who does not have contraindications for surgery. Conservative management has been resorted to in patients with debility with markedly higher mortality.

## Figures and Tables

**Table 1:** 

Laboratory test	Levels	Reference range
White blood cells (WBCs)	4.1 K/CMM	4.5-11.0 K/CMM
Red blood cells (RBCs)	3.77 K/CMM	4.5-6.0 K/CMM
Hemoglobin	10.9 g/dL	13.5 to 17.5 g/dL
Lactic acid	0.9 mmol/L	0.5-2.2 mmol/L
Serum sodium	105 mEq/L	135–145 mEq/L
Serum chloride	74 mEq/L	99-109 mEq/L
Serum osmolality	234 mosm/kg	275–295 mosm/kg
Serum creatinine	0.69 mg/dL	0.40-1.10 mg/dL
Blood urea nitrogen (BUN)	10 mg/dL	5-21 mg/dL
Thyroid-stimulating hormone (TSH)	0.86 mcIU/mL	0.5-5.0 mcIU/mL
C-reactive protein (CRP)	60.5 mg/L	<7 mg/L
Erythrocyte sedimentation rate (ESR)	40 mm/hr	

## References

[1] Umminger J., Krueger H., Beckmann E. (2016). Management of early graft infections in the ascending aorta and aortic arch: a comparison between graft replacement and graft preservation techniques. *European Journal of Cardio-Thoracic Surgery*.

[2] Laohapensang K., Arworn S., Orrapin S., Reanpang T., Orrapin S. (2017). Management of the infected aortic endograft. *Seminars in Vascular Surgery*.

[3] Darouiche R. O. (2004). Treatment of infections associated with surgical implants. *New England Journal of Medicine*.

[4] Patel A., Harris K. A., Fitzgerald F. (2017). What is broad-range 16S rDNA PCR?. *Archives of disease in childhood - Education & practice edition*.

[5] Rossi P., Arata F. M., Salvatori F. M. (1997). Prosthetic graft infection: diagnostic and therapeutic role of interventional radiology. *Journal of Vascular and Interventional Radiology*.

[6] Lawrence P. F. (2011). Conservative treatment of aortic graft infection. *Seminars in Vascular Surgery*.

[7] Lyons O. T. A., Baguneid M., Barwick T. D. (2016). Diagnosis of aortic graft infection: a case definition by the Management of Aortic Graft Infection Collaboration (MAGIC). *European Journal of Vascular and Endovascular Surgery*.

[8] Blanch M., Berjón J., Vila R. (2010). The management of aortic stent-graft infection: endograft removal versus conservative treatment. *Annals of Vascular Surgery*.

[9] Fiorani P., Speziale F., Calisti A. (2003). Endovascular graft infection: preliminary results of an international enquiry. *Journal of Endovascular Therapy*.

